# AAV2-mediated follistatin overexpression induces ovine primary myoblasts proliferation

**DOI:** 10.1186/s12896-014-0087-7

**Published:** 2014-10-21

**Authors:** Mahmood Nazari, Fatemeh Salabi, Li Zhang, Fuping Zhao, Caihong Wei, Lixin Du

**Affiliations:** National Center for Molecular Genetics and Breeding of Animal, Institute of Animal Sciences, Chinese Academy of Agricultural Sciences, Beijing, 100193 People’s Republic of China; Transgenic and Stem Cell Core, Institute of Animal Science, Chinese Academy of Agricultural Sciences, Beijing, 100193 People’s Republic of China

**Keywords:** Follistatin, Over-expression, Adeno-associated virus, Myoblasts, Proliferation

## Abstract

**Background:**

Follistatin (FST) has been shown to bind to some TGF-β family members and can function as a potent myostatin (MSTN) antagonist. Recent studies have revealed that over-expression of FST by adeno-associated viruses increases muscle growth in mice, humans and nonhuman primates. In the present study, to determine the effect of FST on ovine primary myoblast (OPM) proliferation, FST was over-expressed using an adeno-associated virus serotype 2 (AAV 2) vector.

**Results:**

Western blot results showed that AAV induced the expression of FST protein in transduced OPM cells. Real-time quantitative PCR results indicated that over-expression of FST resulted in a dramatic increase in Akt I and CDK2 expression and a decrease in p21 expression. Moreover, cell cycle analysis confirmed that FST down-regulated p21, a CDK inhibitor, and increased the level of CDK2 expression in OPM cells. Hence, follistatin positively regulated the G1 to S progression. Our results showed that FST induced proliferation through a down-regulation of p21, as only the p21 expression level was down-regulated as a result of FST over-expression in myoblasts, whereas no change was observed in the level of p57 expression.

**Conclusions:**

These results expanded our understanding of the regulation mechanism of FST in ovine primary myoblasts. Our results provide the first evidence that the AAV viral system can be used for gene transfer in ovine myoblast cells. Moreover, the results showed that an AAV vector can successfully induce the expression of FST in OPM cells in vitro. These findings demonstrated that FST over-expression induces proliferation through a down-regulation of the p21 gene under proliferating conditions.

## Background

Currently, somatic cell nuclear transfer or pronuclear injection is used to generate transgenic domestic animals. These methods are inefficient, and in some species, they are associated with a high risk of developmental abnormalities in the resulting offspring [1]. Recently, virus-mediated transfer has been employed to generate transgenic animal. Viral vectors provide an alternative, efficient mechanism of delivery. However, there is some controversy regarding the efficiency of transmission of exogenous DNA by viral vectors, such as retroviruses, adenoviruses and lentiviruses [2]. In contrast to retroviruses, recombinant adenovirus-based vectors are able to infect both dividing and non-dividing cells [3]. Adeno-associated viruses are small, nonpathogenic, dependent parvoviruses that can integrate in a site-specific manner into chromosomes [4,5]. The rAAV genome can integrate into the host chromosome, facilitating long-term transduction [6]. Recombinant AAV preparations are stable and can be produced at high titers of more than 10^12^ particles per ml [7]. Adeno-associated viral (AAV) vectors transduce a variety of somatic tissues, including skeletal muscle, without eliciting an immune response in mice [8]. Moreover, recent reports have indicated that adeno-associated virusesare capable of integrating the FST gene into the host chromosome and facilitating long-term transduction [9,10]. Follistatin (FST) has been demonstrated to be a potent antagonist of other members of the TGF-β family, including myostatin [11]. In addition, FST has been shown to be both safe and effective in mice and in nonhuman primates [10]. Studies have also shown that FST is capable of controlling muscle mass through pathways independent of the myostatin signaling cascade [12]. Myostatin (MSTN) negatively regulates myoblast differentiation through activation of the Smad, Akt, p38MAPK and p21 pathways [13-15]. Antagonists of MSTN have shown considerable promise for enhancing muscle mass and strength. MSTN inhibits proliferation and differentiation of myoblasts, limiting the growth rate and muscle mass in mammals [12,16]. Recent studies have highlighted the potential benefit of inhibiting MSTN, which results in a double muscle phenotype in MSTN-deficient cattle [17] and MSTN-knockout mice [18]. In particular, because sheep are an economically important animal, breeding double muscle sheep is of high economic value. However, AAV-mediated FST gene transfer has not been reported in sheep, whereas there are several reports of FST gene transfer in sheep by other vectors, such as lentiviral vectors [19]. The objective of the current study was to use a recombinant adeno-associated virus serotype 2 (rAAV2) carrying follistatin to explore the effects of FST on ovine primary myoblast (OPM) proliferation in vitro. In the present study, we tested the hypothesis that an AAV virus carrying the FST gene is capable of inducing myoblast proliferation in vitro. Additionally, because we recognized that FST is expressed in OPM cells, we verified whether over-expression of FST influences the proliferation of myoblasts irrespective of myostatin. Finally, RT-qPCR analysis was carried out to detect the transcript levels of FST, p21, CDK2, AkT I and ActRII B, which are genes that are thought to play roles that are redundant with MSTN for regulating muscle mass. Our results demonstrated that the FST gene is capable of inducing the proliferation of OPM in vitro. Moreover, the findings indicated that in OPM, over-expression of FST induced proliferation both directly or indirectly through down-regulation of the p21 gene under proliferating conditions.

## Results

### Expression analysis of ovine follistatin and myostatin

**Figure 1:**
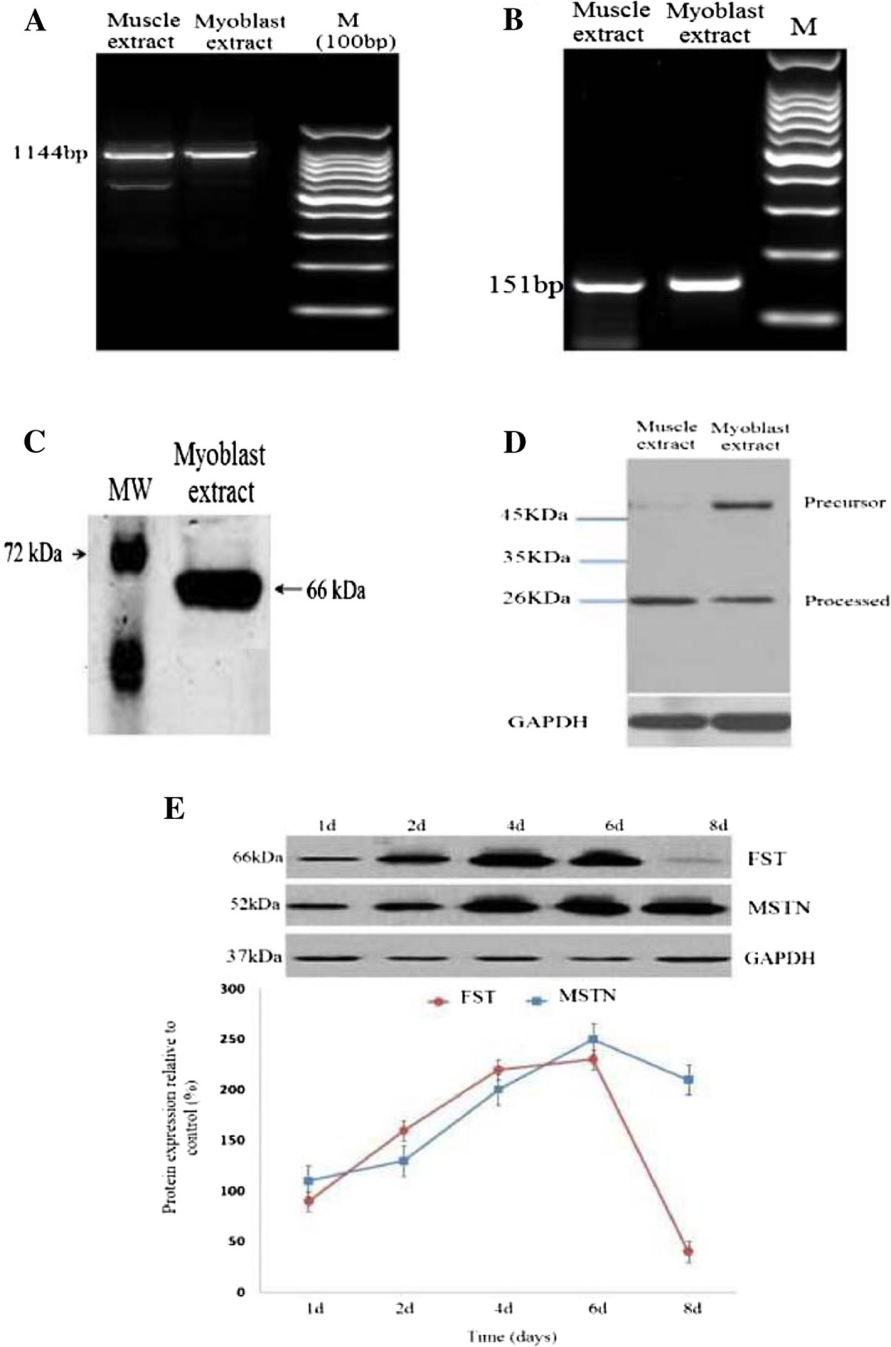
**Detection of follistatin and myostatin mRNA and protein in ovine primary myoblast cells. A**, determination of follistatin mRNA and myostatin. **B**, mRNA expression by RT-PCR in primary muscle myoblasts and ovine fetal skeletal muscle, as a control. M is 100 bp DNA ladder marker. **C**, detection of ovine follistatin protein in primary myoblasts via western blot analysis. 15 μg of total protein was resolved by 12% SDS-PAGE. Polyclonal anti-follistatin (Santa Cruz) was the primary antibody used in the western blot. **D**, detection of myostatin protein in ovine primary myoblast cells and ovine fetal skeletal muscle extracts by western blot analysis. Twenty micrograms of total protein from ovine fetal muscle and 60-day-old ovine primary myoblasts was resolved by 10% SDS-PAGE, and myostatin protein was detected with rabbit anti-myostatin antibodies. Precursor and processed forms of myostatin are indicated. Molecular masses of the western positive bands are indicated. **E**, Densitometry analysis of protein expression for western blots (myostatin and follistatin) was normalized to GAPDH expression in ovine primary myoblast cells over a time-course of 8 days.

To determine whether FST and MSTN mRNA are expressed in OPM, total RNA and protein were isolated from OPM cells and from ovine fetal skeletal muscle to be used as a control and analyzed using RT-PCR. Agarose gel electrophoresis confirmed the expression of FST (Figure 1A) and MSTN mRNA (Figure 1B). The ovine FST and MSTN appeared as a single band on 1.5% (w/v) agarose gels. Electrophoresis of the PCR products showed the 1144 and 151 bp fragments for FST and MSTN, respectively. Next, western blot analyses were performed on protein extracts to detect FST and MSTN protein weight and their properties. Total protein was extracted from OPM cells that were cultured in growth media in 12-well plates for 72 h. The ovine FST protein appeared as a single band with a molecular weight of 66 kDa in SDS–PAGE (Figure 1C). Moreover, anti-MSTN antibodies specifically recognized the two following bands: precursor and processed forms of MSTN (Figure 1D). These two bands corresponded to the unprocessed full-length protein (52-kDa) and the mature processed MSTN (26-kDa). However, this 52-kDa precursor myostatin was not detectable in muscle extracts. These results indicated that OPM cells are capable of synthesizing FST and MSTN mRNA and protein in vitro.

To confirm the RT- qPCR results, FST and MSTN protein expression were also measured by western blot analysis for OPM cultured in GM over an 8-day time-course proliferation. Western blot results indicated that MSTN and FST protein expression was up-regulated during proliferation. FST protein expression was decreased at day 8, whereas MSTN expression remained constant at the end of culture period (Figure 1E).

### Cloning of ovine follistatin

**Figure 2:**
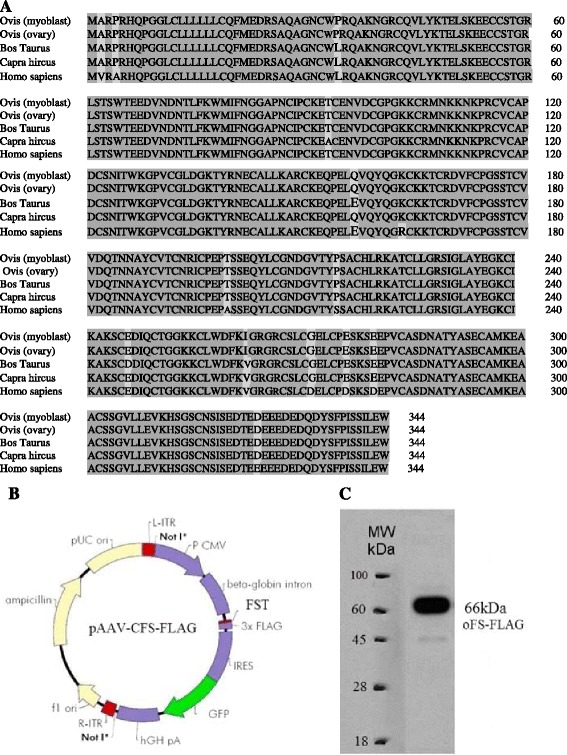
**Box shade diagram showing similarity of deduced amino acid sequences encoding ovine follistatin in primary myoblast cells. A**, Multiple sequence alignment of proteins was carried out using the DNAMAN 6.0 method based on amino acid sequences of follistatins from Ovisaries (NP_001244022.1), Bos Taurus (NP_786995.2), Capra hircus (ADJ53355.1) and Homo sapiens (BC004107.2). **B**, Gene map of recombinant plasmid pAAV-CFS-FLAG: ITR: inverted terminal repeat; CMV promoter: Cytomegalovirus promoter; FST: coding region for follistatin; IRES: internal ribosome entry site; GFP: green fluorescent protein; hGHpA: human growth hormone (hGH) polyA signal; **C**, Detection of FST-FLAG proteins in transduced and normal ovine primary myoblasts by western blot analysis. 15 mg of total protein was resolved by 12% SDS-PAGE. Monoclonal anti-Flag (Sigma) were the primary antibody used in the western blot.

The full-length sequence of the gene encoding FST in OPMs, containing a 1035 bp segment encoding the 344 amino acid, was cloned into a pGEM-T vector and sequenced (GenBank Accession No. KF833357). From the follistatin CDS sequence, the amino acid sequence was deduced using software from NCBI. Multiple alignments of the amino acid sequences were produced via DNAMAN 6.0, as shown in Figure 2A. The deduced amino acid sequence of the ovine FST is highly homologous to amino acid sequences of follistatins from ovine (*Ovisaries*, NP_001244022.1, 100%), goat (*caprahircus*, ADJ53355.1, 99%), bovine (*Bos Taurus*, NP_786995.2, 99%) and human (*Homo sapiens*, NP_037541.1, 92%) ovaries.

### Follistatin protein is synthesized in transduced myoblast cells

Construction of the expression vector is shown in Figure 2B. FST cDNA was subcloned into pAAV-IRES-GFP, resulting in the recombinant expression plasmid pAAV-CFS-FLAG (Figure 2B), which was verified via DNA sequencing. Our results confirmed the expression of ovine FST in the transduced OPM cells. As shown in Figure 2C, anti-flag monoclonal antibodies specifically recognized a 66-kDa band for ovine FST in the transduced OPMs. The result here for recombinant FST driven by CMV promoter in transduced cells was similar with observed band in Figure 1C for endogenous FST.

### Changes in mRNA concentration during myoblast proliferation

**Figure 3:**
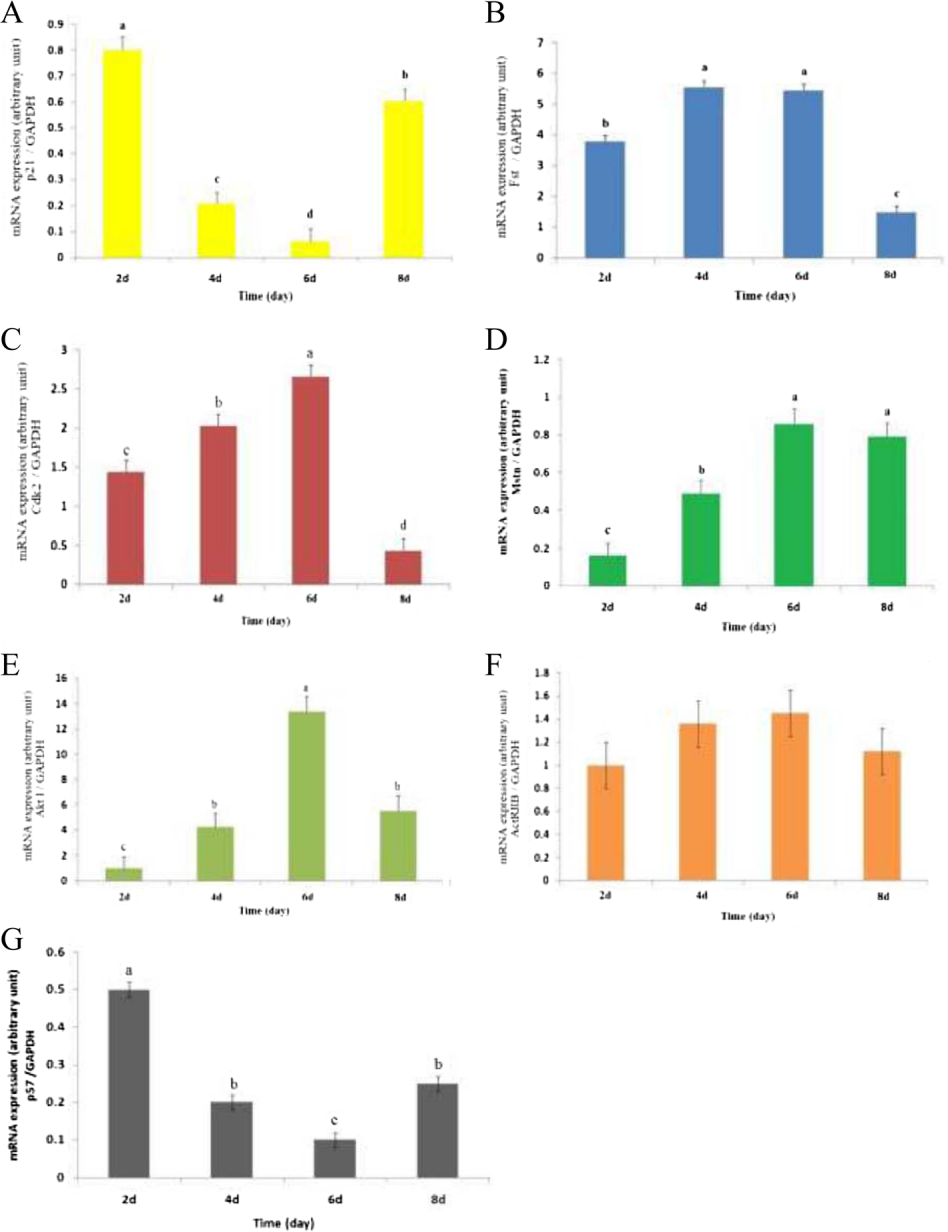
**Real time quantitative PCR results of p21 (A), FST (B), CDK2 (C), MSTN (D), Akt I (E), ActRIIB (F)and p57 (G) mRNA expression during proliferation of ovine primary myoblast.** Cells cultured in 6-well plates for eight days and harvested at days 2, 4, 6 and 8. The mRNA concentrations were quantified by real-time quantitative PCR. The amount of mRNAs was normalized to the amount GAPDH mRNA. Data were analyzed by one-way ANOVA (P < 0.05, n = 8). Statistical differences are indicated: a > b > c > d.

To characterize the growth rate of the OPM cells, the cells were seeded at 2 × 10^4^ cells/well in 6-well plates and harvested and counted for eight days. The result indicated that the cells entered the logarithmic phase of growth after 2 days and grew extremely slowly after 6 days. Some fused cells were observed after 6 days. Eight days after incubation, many of the cells were fused and had a long tubular shape (data not shown). Next, to gain insight into the role of follistatin during OPM proliferation and to assess the expression of p21, p57, Act RIIB, Akt I, CDK2, MSTN and FST genes during OPM proliferation, total RNA was isolated from the OPM cells and harvested at days 2, 4, 6 and 8. In addition, the cells were harvested at day 1 to be used as a control. Then, the mRNA concentration was quantified by RT-qPCR. We noted that there were no significant changes in Act RIIB mRNA expression (Figure 3F). As shown in Figure 3, FST, MSTN, Akt I and CDK2 mRNA concentrations were up-regulated during OPM proliferation. The amounts of these genes gradually increased at day 2 (P <0.01) after plating and then sharply increased up to day 6. FST, Akt I and CDK2 mRNA levels declined at day 8 (P <0.01) after plating, whereas the level of MSTN mRNA remained constant until the end of the culture period. As shown in Figure 3, the maximum expression levels of FST and CDK2 were observed at day 6. Both genes began to show increased expression after 2 days, reaching a maximum expression at day 6, and exhibited low expression at day 8. Moreover, the results showed that the p21 and p57 mRNA concentrations were down-regulated during myoblast proliferation (Figure 3A). Interestingly, the minimum expression levels of p21 and p57 were observed at day 6 (Figure 3A). However, the levels of p21 and p57 mRNA were both increased at day 8, which paralleled the increase in MSTN mRNA concentration and the reduction in FST mRNA concentration at day 8. Taken together, these findings strengthened the hypothesis that FST may be involved in the proliferation of myoblasts.

### Effects of ovine FST over-expression on gene expression

**Figure 4:**
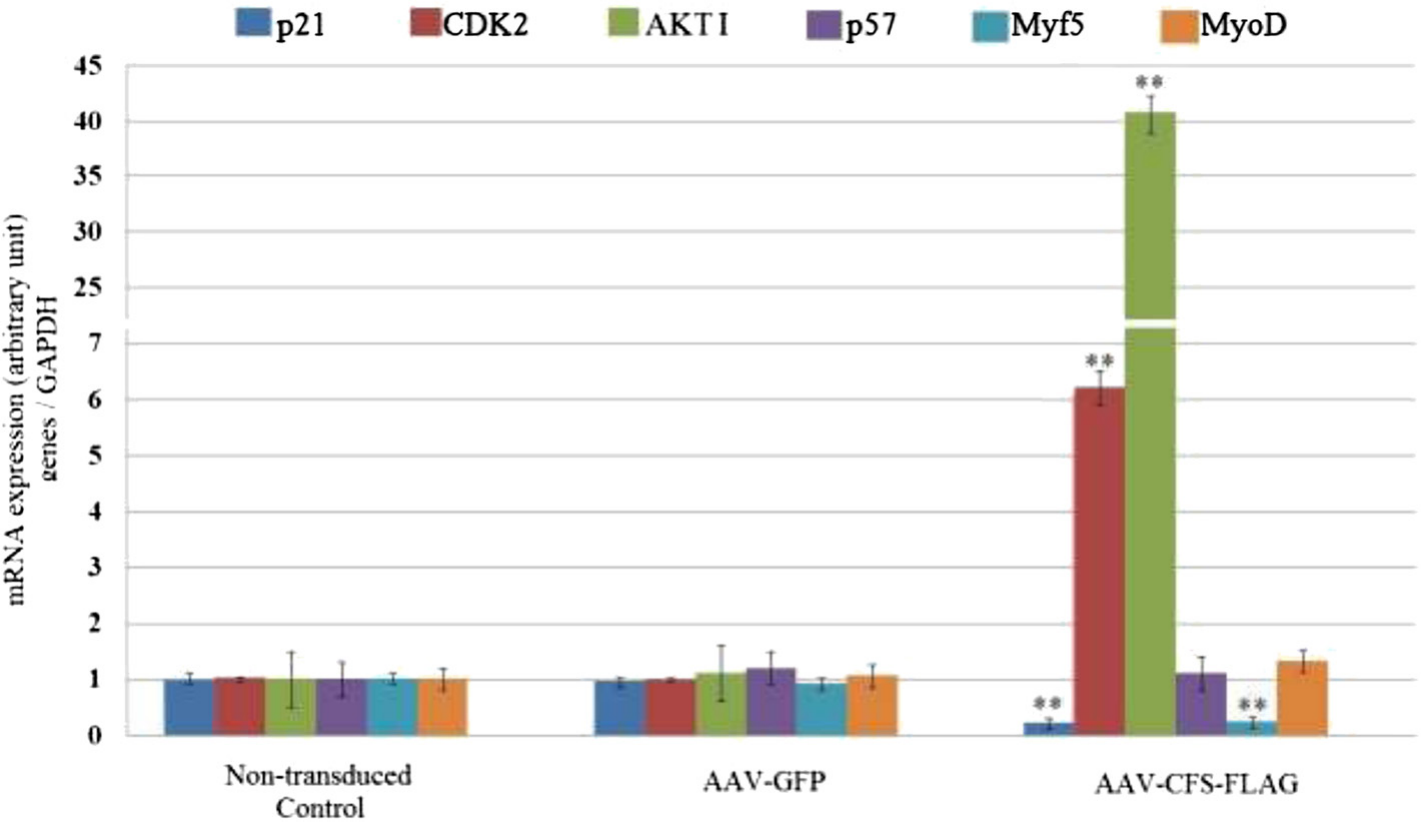
**Real time quantitative PCR results of p21, MyoD, Akt I, CDK2, p57 and Myf5 genes expression in transduced primary myoblasts cultured under proliferating conditions.** Total RNA was isolated after 96 h from the OPM transduced with AAV-CFS-FLAG and the non-transduced OPMs (control). Gene expression was determined using real-time qPCR and normalized to GAPDH mRNA. The non-transduced myoblasts and AAV-GFP were selected as the control and negative control, respectively. **, P < 0.01 with non-transduced control.

In this experiment, total RNA was extracted from the transduced OPM to detect p21, p57, Myo D, Akt I, CDK2 and Myf5 gene expression using RT-qPCR under proliferating conditions. We noted that AAV-CFs-FLAG exhibited a 12.5-fold reduction in p21 mRNA expression (P < 0.01). Moreover, the expression of CDK2 exhibited a 6.1-fold increase (P < 0.01) compared to the control. In addition, Akt I mRNA expression exhibited a dramatic 41-fold increase (P < 0.01).These data also revealed that the expression of Myf5 mRNA exhibited a 2.5-fold decrease (P < 0.01). In addition, there were no significant changes in Myo D and p57 mRNA concentrations compared to the control (Figure 4).

### Follistatin over-expression increases proliferation of ovine primary myoblasts

**Figure 5:**
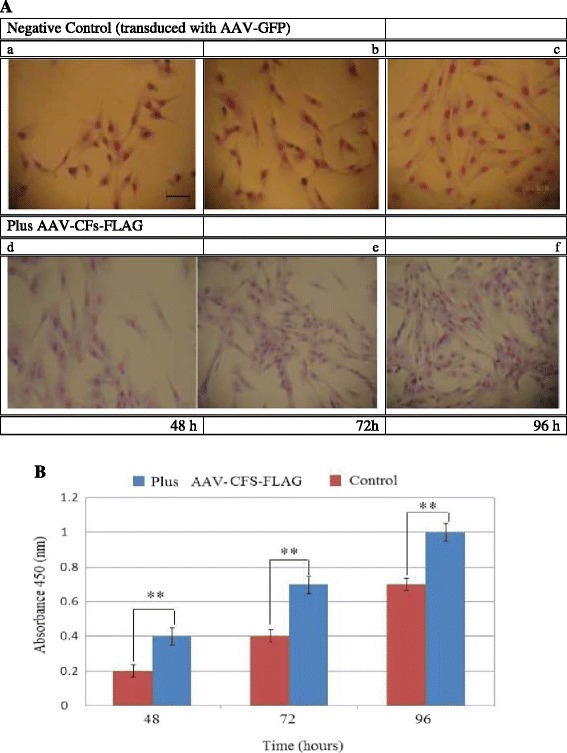
**Induction of ovine primary myoblast proliferation by AAV-CFs-FLAG virus expressing FST protein in vitro. A**, phase contrast pictures show the morphology of ovine primary myoblast cells after follistatin over-expression, as compared to negative control cells. After Giemsa staining, pictures were taken under the same magnification over a time-course of 4 days. Myoblasts were grown for 48, 72 and 96 h in growth media without or with AAV-CFs-FLAG virus transduction. Cells were fixed and stained with Giemsa. Bar =100 μM. **B**, absorbance (at 450 nm) was read at 48, 72 and 96 hours after plating in growth media without or with AAV-CFs-FLAG virus. Data were analyzed by one-way ANOVA (**P <0.01, n = 8).

To determine the effect of ovine FST protein on OPM cell proliferation, we cultured OPM cells for 48, 72 and 96 h in growth media infected with or without the AAV-CFs-FLAG virus. The cells were fixed and stained with Giemsa (Figure 5A, a–f). OPM proliferation was monitored for alterations using a WST-8 assay with Cell-Counting Kit-8 (CCK-8) reagents. As shown in Figure 5B, the optical density (at 450 nm) was significantly increased in the transduced OPMs cultured in GM media after 48, 72 and 96 h compared with the control, indicating that FST significantly increased proliferation (*P*< 0.01). However, the FST protein induced the growth of myoblasts in a time-dependent manner with half-maximal induction occurring at the 72 h time point (Figure 5B). In addition, FST did not cause an increase in myotube formation and overt differentiation (Figures 5A, a–f).These data indicated that when OPM cells were incubated in GM with the AAV-CFs-FLAG virus for 96 h, there was a steady increase in cell number compared to the control (Figure 5A, a–f). The results strongly suggested that AAV-CFs-FLAG-expressing FST induced a significant effect on cell myoblast proliferation, suggesting that it is involved in modulating myoblast proliferation.

### FST protein interferes at the G1 phase of the cell cycle

**Figure 6:**
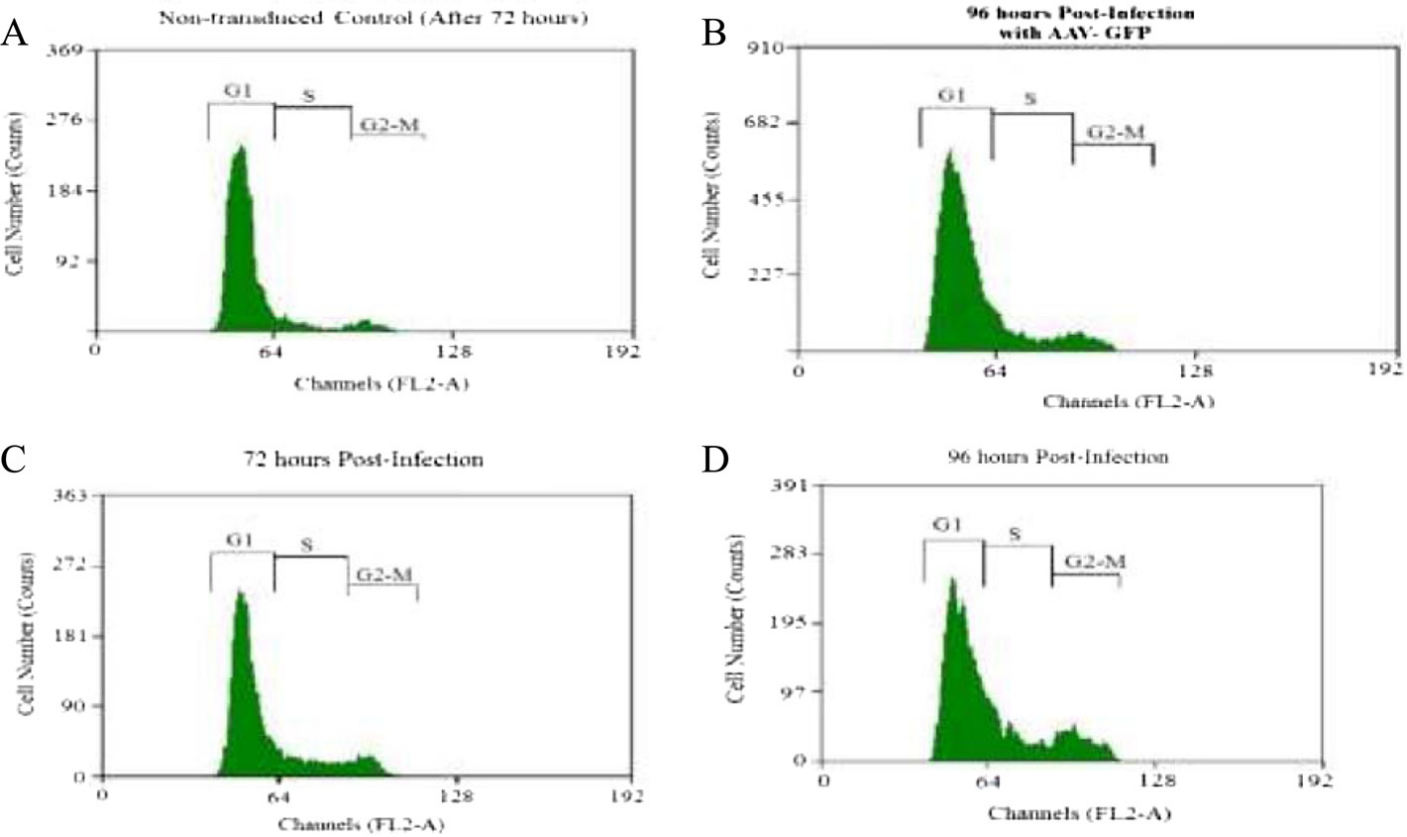
**Effect of follistatin over-expression on ovine primary myoblast cell cycle progression. A**, cell cycle distribution was measured by flow cytometry using propidium iodide stain and the percent of the cell cycle phase is shown in a bar graph form with the G1 (85.45%) and S (9.23%) phases. **B**, cell cycle distribution was measured by flow cytometry and the percent of the cell cycle phase is shown in a bar graph form with the G1 (81.65%) and S (11.49%) phases. **C**, cell cycle distribution was measured by flow cytometry using propidium iodide stain and the percent of the cell cycle phase is shown with the G1 (73.93%), and S (16.24%). **D**, cell cycle distribution was measured by flow cytometry and the percent of the cell cycle phase is shown with the G1 (63.43%), and S (27.10%).

The cell cycle was analyzed using a fluorescence-activated cell sorter (FACS) after staining with propidium iodide (Figure 6). The cells were transduced in growth medium for 72 and 96 h. The percentage of cells in the G1, S, and G2/M phases was significantly different under the various conditions (*P*< 0.05). The detailed values of the cell cycle analysis are summarized in Table 1. As shown in Table 1, the percentage of cells in G1 was significantly decreased and the percentage in S phase increased with AAV-CFs-FLAG transduction. The FACS analysis also showed a dramatic increase in the number of cells in S phase (9.23 ± 1.60% to 27.10 ± 1.81%), which was accompanied by a decrease in the percentage of cells in G1 (85.45 ± 2.12% to 63.43 ± 1.95%) phase of the cell cycle (Figure 6; Table 1). In addition, there were no significant changes in AAV-GFP during the G1 and S phases compared to non-transduced cells (Figure 6B). Furthermore, AAV-CFs-FLAG transduction reduced the G1 phase in a time-dependent manner, with the maximum reduction at 96 h post-infection (Figure 6D). These data suggest that follistatin reduced the G1 phase in growth medium. The results confirmed that the increase in myoblast proliferation was due to a decrease in the number of cells in G1 phase (Figure 6; Table 1).

**Table 1:** **The percentage of cells in G1, G2/M and S-phases under different conditions (mean ± S.E.M)**

**Conditions**	**G1 (%)**	**G2 /M (%)**	**S (%)**
Non-transduced control (after 96 h)	85.45 ± 2.12^a^	5.32 ± 0.87^b^	9.23 ± 1.60^c^
96 hours Post-Infection with AAV-GFP	81.65 ± 2.41^a^	6.86 ± 1.12^ab^	11.49 ± 1.34^c^
72 hours Post-Infection with AAV-CFs-FLAG	73.93 ± 1.85^b^	9.83 ± 0.56^a^	16.24 ± 1.45^b^
96 hours Post-Infection with AAV-CFs-FLAG	63.43 ± 1.95^c^	9.47 ± 0.93^a^	27.10 ± 1.81^a^

Cell cycle analysis was performed at 72 and 96 hours after those cells was transduced with AAV-CFs-FLAG and the percentage of cells in G1, G2/M and S-phase were determined. The percentage of cells in G1 phase was greatly significant 96 hours AAV post-infection. Data were analyzed via one-way ANOVA (*P* < 0.05). Statistical differences are indicated: a > b > c.

## Discussion

Recent studies have shown that human myoblast proliferation is induced by an over-expression of FST in cell culture. In addition, FST forms a complex with MSTN, preventing its interaction with its receptor and thus blocking the MSTN signal [20]. In the present study, ovine FST was cloned into the AAV2 vector, and then AAV2 virus particles of high titer were obtained. The OPM cells were infected by recombinant AAV vectors. After total RNA and protein isolation, real-time qPCR and western blotting were performed for the OPMs cultured in the GM. Furthermore, a cell proliferation assay was performed using Cell-Counting Kit-8 (CCK-8) reagents. The cells were stained with propidium iodide and were analyzed using a flow cytometer for cell cycle progression. The results in Figure 1 show that the amino acid sequence deduced from the cloned full-length DNA sequence of FST contained an N-terminal domain (residues 1 ~ 63), domain 1 (64 ~ 136), domain 2 (137 ~ 211), domain 3 (212 ~ 288), and a C-terminal region (288 ~ 315). Therefore, the sequence indeed encoded the longest form of FST [21]. The western blot analyses showed that the ovine FST protein appeared as a single band with a molecular weight of 66-kDa in SDS–PAGE (Figure 2B), consistent with previous studies [19]. In this study, we observed the precursor and the processed MSTN at 52 and 26-kDa proteins, respectively (Figure 2F). These observations were consistent with previous reports [16,22-24]. These results indicated that the MSTN protein was indeed synthesized and proteolytically processed in the OPMs.

To understand the regulation mechanism of MSTN and FST protein and their interaction, real-time qPCR analysis was performed on the OPM cells undergoing proliferation. Moreover, to confirm the RT- qPCR results, FST and MSTN protein expression were also measured by western blot analysis. FST and MSTN mRNA concentrations were up-regulated at mRNA (Figure 3B and D) and protein level (Figure 1E), whereas p21 and p57 mRNA concentrations were down-regulated during OPM proliferation (Figure 3A and G). Despite the increased expression of MSTN mRNA, p21 and p57 mRNA expression was decreased in this condition. It has been thought that increased expression of MSTN leads to an up-regulation of p21 and p57 mRNA expression [23]. The lack of consistency demonstrated that the FST protein, as a binding protein for MSTN, may prevent MSTN from binding to receptors on the myoblast cells and thus block the MSTN signal [20]. However, the RT-qPCR results showed that FST over-expression only down-regulated p21 mRNA expression and had no effect on p57 mRNA expression. These results confirmed that FST induces proliferation through a down-regulation of p21 because only p21 levels were down-regulated as a result of FST over-expression in myoblasts (Figure 4), whereas no change was observed in the level of p57 expression [23].

Because FST can lead to increased muscle mass [9], it is possible that FST functions by increasing the number of myoblasts during development. To examine this possibility, we incubated myoblast cultures with an AAV vector carrying the FST gene. The results indicate that FST indeed induces the proliferation of exponentially growing myoblasts (Figure 5A). Moreover, because the surviving myoblasts at the end of the assay reflect the net result of cell proliferation, we performed a flow cytometry analysis to assess the cell cycle progression in the transduced myoblasts. The flow cytometry analysis showed that when myoblasts were transduced with the AAV-CFS-FLAG virus, a decreased number of myoblast cells was arrested inG1phase, and therefore, more myoblast cells made the transition to S (DNA synthesis) phase (Figure 6; Table 1). Consistent with this finding, others have suggested that MSTN can inhibit myoblast proliferation [23,25,26]. Investigations into the mechanisms revealed that one of the targets of MSTN signaling is the cell cycle. In cell culture studies, MSTN inhibited the progression of myoblasts at the G1 phase of the cell cycle. This cell cycle arrest was shown to be mediated by p21, which was the only CDK inhibitor to be up-regulated by MSTN. In addition, myostatin treatment also specifically reduced the levels of CDK2, the combination of which resulted in markedly reduced activity of CDK2. This, in turn, led to the hypophosphorylation of retinoblastoma protein and thus inhibited the progression of cells into the S phase of the cell cycle [23]. Here, in the OPMs infected with AAV-CFs-FLAG, there was indeed a decrease in p21 expression at the transcription level and an increase in CDK2 expression (Figure 4) under proliferating conditions, which led to a decrease in the arrest of myoblasts in the G1-phase of the cell cycle. FST likely forms a complex with MSTN, preventing its interaction with its receptor and thus blocking the MSTN signal [20]. Thus, the observed increase in the number of myoblast cells is the result of deregulated (increased) myoblast proliferation. These observations are consistent with the model proposed by Thomas et al. [23] regarding the role of MSTN during muscle growth [23]. We propose the following model for the role of FST in myoblast proliferation regulation. In response to MSTN signaling, consistent with the up-regulation of p21, CDK2 expression is down-regulated, which causes the hypophosphorylation of the Rb protein and G1 arrest. Therefore, these results confirmed the role of FST in myoblast proliferation regulation.

## Conclusions

Our results provide the first evidence that an AAV viral system can be used for gene transfer in sheep. Moreover, the results showed that an AAV vector can successfully induce expression of FST in OPM cells in vitro. In addition, our findings demonstrated that FST induces proliferation through a down-regulation of the p21 gene under proliferating conditions in ovine primary myoblasts. In the future, it would be interesting to determine whether an AAV virus can be used to produce transgenic sheep for meat production.

## Methods

### Nucleic acid manipulation

DNA was purified and manipulated, as described previously [27].

### Chemicals, strains, plasmids and media

T4 deoxyribonucleic acid (DNA) ligase and restriction endonucleases were purchased from New England Biolabs (Beverly, MA) and Fermentase (Life Sciences, Thermo Fisher), respectively. Escherichia coli DH5 alpha (Invitrogen, CA, USA) was used as a host for the plasmid cloning experiments. Bacteria were grown in Luria-Bertani (LB) medium containing 5 g/L yeast extract, 10 g/L tryptone and 10 g/L NaCl at pH = 7.0 and 37°C. The pAAV-IRES-GFP plasmids, consisting of a CMV promoter and a FLAG tag, the pAAV-GFP control, and the pAAV-RC and pHelper vectors were purchased from Agilent Technologies Company (La Jolla, CA). The pGM-T plasmid vector for the direct insertion of a blunt PCR product into a plasmid vector was obtained from Invitrogen Company (CA, USA). Plasmids were amplified in DH5 alpha bacteria, purified (Gene JET Plasmid Maxiprep Kit; Fermentas, Life Sciences, Thermo Fisher) and dissolved in nuclease-free water. Dulbecco’s Modified Eagle’s Medium (DMEM) was supplemented with 100 U/ml penicillin and 100 μg/ml streptomycin. Fetal bovine serum (Hyclone, Logan, UT) and horse serum (Hyclone, Logan, UT) were also added to DMEM to prepare specific media.

### Cloning of the full-length follistatin sequence

Ovine full-length FST cDNA was isolated from a primary myoblast cDNA library, and its sequence was determined. To detect the full-length FST cDNA sequence, the total RNA was extracted from OPMs using a GeneJET RNA Purification Kit (Fermentas, Life Sciences, Thermo Fisher). Then, the cDNA was synthesized using a Maxima First Strand cDNA Synthesis Kit (Fermentas, Life Sciences, Thermo Fisher) according to the manufacturer’s protocol. The DNA encoding FST was amplified via PCR from cDNA using the forward primer 5′-GCACCGCTTGCCAGAGCCAC -3′ and the reverse primer 5′-GGTGGAGGGTTTACCACTCTAGAATGGA -3′, which were designed based on the availability of bovine sequences (Gen Bank Accession No. NM_175801). The PCR reaction was carried out in a final volume of 20 μl containing the DNA template and 10 μl of commercially available PCR master mix (PyroStart™ Fast PCR Master Mix (2×), Fermentas, Life Sciences, Thermo Fisher). After brief centrifugation, the PCR amplification was carried out in an ABI 9700 PCR system (Applied Biosystems, Foster City, CA, USA). The conditions for the PCR amplification were as follows: initial denaturation was performed at 94°C for 4 min, followed by 35 cycles at 95°C for 30 s, 60°C annealing for 30 s, and 72°C extension for 1 min, and a final extension at 72°C for 10 min. The amplified DNA was ligated into a pGM-T vector and transformed into DH5a (E. coli). The DNA sequence was determined after digestion by a restriction enzyme, electrophoresis, and sequencing. The amino acid sequence of the cloned DNA sequence was determined using software from NCBI. Multiple alignments of the amino acid sequence were produced using DNAMAN 6.0.

### Construction of the pAAV-CFS-FLAG plasmid vector

A complete open reading frame (ORF) of 1035 base pairs of the ovine FST gene was amplified via PCR from full-length cDNA using the forward primer 5′-AA**GAATTC**CCTCAGGATGGCCCGTCCTA-3′ (P1) containing an EcoRI site (bold) and the reverse primer 5′-G**CTCGAG**GGTTTTCCACTCTAGAATGGA -3′ (P2) containing an XhoI site (bold). The primers were designed based on the availability of ovine sequences (Gene Bank Accession No. KF833357). The PCR reactions and conditions were carried out as described above. The PCR products and the pAAV-IRES-GFP vector (Agilent Technologies Company, La Jolla, CA) were digested with EcoRI and XhoI (Fermentas, Life Sciences, Thermo Fisher), recovered through agarose gel electrophoresis, and then ligated by T4 DNA ligase (New England Biolabs, Beverly, MA). New recombinant plasmids, which consisted of a CMV promoter and a FLAG tag, carrying the ovine FST gene were termed pAAV-CFS-FLAG. The ligated plasmid vector was transformed into E. coli DH5 alpha. A single colony of the transformed bacteria was selected and transferred to 3 ml of Luria-Bertani (LB) medium containing 100 μg/mL ampicillin and incubated overnight under vigorous shaking (200 rpm) at 37°C. The culture was used to extract the recombined plasmid. The presence of the FST protein was confirmed via digestion with a restriction enzyme, electrophoresis, and sequencing.

### Packaging AAV vectors

AAV particles were produced by co-transfection of the recombinant pAAV-CFS-FLAG vector (20 μg) with two helper plasmids (20 μg each of pAAV-RC and pHelper) into HEK 293 cells seeded in a 225-cm^2^ flask. Briefly, 1 h before transfection, half of the medium was replaced with fresh medium, and 20 μg of vector and of each helper plasmid was added to 4 ml of 300 mM CaCl_2_. This solution was added to 4 ml of 2× HBS and mixed immediately by gently inverting three times. The mixture was immediately pipetted into HEK 293 cells in 40 ml of DMEM medium with 10% FCS. The plate was incubated at 37°C for 6 h. Three days after transfection, 1 ml of 0.5 M EDTA was add and incubated for 3 min at room temperature. The supernatant was collected at 24, 48, and 72 h via centrifugation at 1000 × g for 10 min at 4°C. Next, the supernatant was removed and the cells of the pellet were resuspended in 2 ml of TBS. The cells were freeze-thawed that had been suspended in TBS three times by placing them alternately in a dry ice/ethanol bath until the suspension was completely frozen and in a water bath of 37°C until it was completely thawed. Then, the tissue debris was removed via centrifugation at 20,000 g for 10 min, and the supernatant was collected. The collected supernatant was filtered through a 0.45-μm low protein binding filter (Millipore, Billerica, MA, USA). Finally, a ViraBind™ Adeno-Associated Virus Purification Mega Kit (Cell Biolabs, INC, San Diego, CA, USA) was used according to manufacturer’s protocol. The titer of the virus concentrate was determined by transducing 293 cells with serial dilutions. The suspended virus particles were diluted using serial dilutions of 10^−1^, 10^−2^, 10^−3^, and 10^−4^. After culturing for 48 h, the titers were determined by counting the number of GFP-positive colonies using a fluorescence microscope. The titers were expressed as transducing units/mL (TU/mL). The virus preparation used for transgenesis had a final titer of greater than 10^12^ transducing units/mL.

### Ovine primary myoblast culture

Primary myoblast cultures were obtained from 60-day-old sheep fetuses, as previously described [28]. Briefly, hind limb skeletal muscle mass was excised, cut into small pieces, placed in DMEM containing 20% FBS and 10% Me2SO, and frozen in liquid nitrogen for subsequent myoblast cultures. Primary muscle cultures were grown in growth media (DMEM with 10% FBS, 100 U/mL penicillin and 100 μg/mL streptomycin) in 6-cm petri dishes to test for fibroblast contamination. Preliminary studies (data not shown) indicated that approximately 90% of the ovine primary myoblast cells stained positive for the muscle-specific marker desmin. All studies were approved by the Biological Studies Animal Care and Use Committee, Beijing, Peoples Republic of China.

### Transduction of primary myoblast cells

For transduction of primary myoblasts with AAV-CFs-FLAG or AAV-GFP control vectors, a ViraDuctin™ AAV Transduction Kit (Cell Biolabs, INC, San Diego, CA, USA) was used according to the manufacturer’s instructions as the transduction reagent. Briefly, the OPM cells were seeded at 3 × 10^3^ cells/well in 48-well plates. An AAV virus with the optimal multiplicity of infection (MOI) of 50 was added to the fresh growth medium. The cells were incubated at 37°C for 6 h. Finally, pre-warmed complete medium was added in each well and incubated at 37°C for 48, 72 or 96 h. The green fluorescence excited by the AAV vector was observed using a fluorescence microscope (ECLIPSE TE2000-U, Nikon, Tokyo, Japan) at 48, 72 or 96 h.

### RNA isolation and RT-PCR

The total RNA was isolated from primary myoblast cells and ovine fetal skeletal muscle (20 mg) using a Thermo Scientific GeneJET RNA Purification Kit (Fermentas, Life Sciences, Thermo Fisher) according to the manufacturer’s instructions. The quality of RNA was verified using a spectrophotometer (Astra Gene, Astra net, Cambridge, USA) at 260/280 nm (OD260/OD280 = 1.8–2.0). The cDNA was prepared from 1 μg of total RNA using a Maxima First Strand cDNA Synthesis Kit (Fermentas, Life Sciences, Thermo Fisher) according to the manufacturer’s protocol. The sequences of primers, amplicon size and annealing temperature are shown in Table 2. The PCR reactions and conditions were carried out as described above.

**Table 2:** **Primers used for RT-PCR and real time quantitative PCR**

**Gene**	**Primer sequence (5′–3′)**	**Anneal. T (°C)**	**Amplicon size**	**GenBank accession number**
Myostatin	F: 5′-CTGGCCCAGTGGATCTGAATGAG −3′	60	151 bp	NM_001009428
	R: 5′- TTCCAGGCGAAGCTTACTGAGGA-3′			
Follistatin	F: 5′-TGGACCGAGGAGGACGTGAA-3′	60	160 bp	NM_001257093.1
	R: 5′-AGACGCAGCGGGGTTTGTTC-3′			
ActRIIB	F: 5′-TCCCTCACGGATTACCTCAAG-3′	58	149 bp	NM_174495
	R: 5′-TCCCTGTGGGCAATAGATGG-3′			
Myf5	F: 5′-ACCAGCCCCACCTCAAGTTG-3′	60	150 bp	NM_174116.1
	R: 5′-GCAATCCAAGCTGGATAAGGAG-3′			
MyoD	F: 5′-CGACTCGGACGCTTCCAGT-3′	60	181 bp	NM_001040478.2
	R: 5′-GATGCTGGACAGGCAGTCGA-3′			
P21	F: 5′-GCAGACCAGCATGACAGATTT-3′	58	70 bp	NM_001098958
	R: 5′-GGATTAGGGCTTCCTCTTGGA-3′			
P57	F: 5′- CGCCGCACCTTTCCCATG-3′	59	166	NM_001142510
	R: 5′- CCCGCAGTGGCATGTCCA-3′			
CDK2	F: 5′-CATGGATGCCTCTGCACTCACTG-3′	60	102 bp	NM_001142509.1
	R: 5′-AGGACCCGATGAGAGTGGCAGAA-3′			
Akt1	F: 5′-CAGCATCGTGTGGCAGGAC-3′	60	138 bp	NM_001161857.1
	R: 5′-TCTTGGTCAGGTGGCGTAA-3′			
GAPDH	F: 5′-CAAGTTCCACGGCACAGTCA-3′	60	249 bp	NM_001190390
	R: 5′-TGGTTCACGCCCATCACAA-3′			

### Real-time quantitative RCR analysis

Myoblast cells (3.0 × 10^3^ cells/well) were cultured in GM in 48-well plates. Total RNA was isolated after 96 h from the OPM transduced with AAV-CFS-FLAG and the non-transduced OPMs (control). Moreover, an AAV-GFP control vector was also selected as a negative control for these experiments. The mRNA concentrations for all of the genes, which are listed in Table 2, were quantified via real-time quantitative PCR (RT-qPCR). Primer sequences were designed using the Vector NTI Advance™ 11. The sequences of primers for PCR amplification are shown in Table 2. In this procedure, GAPDH was used as a house-keeping gene for normalizing the gene expression data of the RT-qPCR. RT-qPCR was performed using a BIO-RAD iQ5 Real-time PCR Detection System (Bio-Rad, Hercules, CA, USA) with a Power SYBR-Green PCR Master Mix Kit (Applied Biosystems, Foster City, CA, USA). RT-qPCR was carried out with a final volume of 20 μL containing 1 μL RT product, 10 μL SYBR-Green PCR master mix (2×), 2.5 μM forward and reverse primers and nuclease-free water. The PCR cycling programs were as follows: 3 min of UNG incubation at 55°C, followed by 10 min at 95°C, 40 cycles at 95°C for 10 s, annealing at an optimized annealing temperature for 15 s (temperatures given in Table 2), extension at 72°C for 15 s, plate-reading every 0.2°C from 60°C to 94°C for calculation of the melting curves, and a final extension step of 72°C for 10 min. Finally, fluorescence data were analyzed using the iQ5 Optical System Software (Bio-Rad). Gene expression was normalized to GAPDH expression using the 2^–ΔΔC^_T_ method [29].

### Western blot analysis

Total protein was extracted from the normal myoblast cells and the transduced myoblast cells to determine MSTN and FST protein expression. Total protein was obtained from hind limb fetal skeletal muscle as a control. Protein concentrations were determined according to the manufacturer’s instructions (Bio-Rad, Hercules, CA, USA). A total of 15 μg protein was separated via SDS-PAGE (12%) and transferred to a nitrocellulose membrane via electro-blotting. SDS-PAGE and western blot transfer were performed using standard techniques. The gels run under non-reducing conditions. The following primary antibodies were used in this study: MSTN, goat polyclonal anti-MSTN antibody (sc-6884, 1:500; Santa Cruz); FST-FLAG, mouse monoclonal anti-FLAG M2 antibody (F-3165; 1:500; Sigma); FST, rabbit polyclonal anti-FST antibody (sc-30194, 1:500; Santa Cruz) and GAPDH, mouse monoclonal anti-GAPDH antibody (G8795, 1:5000; Sigma). HRP-conjugated goat anti-mouse IgG antibody (115-035-174, 1:10,000; Jackson Immuno Research, West Grove, PA, USA), rabbit anti-goat IgG-HRP (sc-2922, 1:5000; Santa Cruz) and HRP-conjugated goat anti-rabbit IgG antibody (111-035-003, 1:10,000; Jackson Immuno Research) were used as secondary antibodies.

For myostatin protein detection, protein (20 μg) was loaded for electrophoresis on a 10% gel and transferred to nitrocellulose membrane (Millipore, Billerica, MA, USA) by wetting transferring. Membranes were stained with Ponceau stain to verify loading. Membranes were blocked in 3% BSA-TBST and incubated overnight at 4°C with primary antibodies. Then, Membranes washed five times for 5 min each time with TBST, and incubated for 60 min at room temperature with secondary antibodies.

For GAPDH-loading controls, the membrane was blocked in 3% BSA-TBST and incubated with anti-GAPDH antibody in 3% vBSA-TBST for 3 h at room temperature, followed by incubation with secondary antibody in TBST +5% milk for 60 min at room temperature. Horseradish peroxidase activity was detected using ECL with western blotting reagents (RPN2132, Amersham Biosciences). Finally, the protein expression quantified by densitometry by the Gel Quant NET software, and normalized to GAPDH.

### Proliferation assay

The OPM cells were grown prior to the assays in growth media. Cell proliferation assays were conducted in 48-well plates. After a 16-h attachment period, the cells were transduced with the AAV-CFs-FLAG vector. The plates were then incubated at 37°C and 5% CO2 for48, 72 and 96 h. After the incubation period, the cell proliferation assay was performed by adding 20 μl of the Cell-Counting Kit-8 (CCK-8) reagents (Dojindo Molecular Technologies, Maryland, USA) to each well of the plate for 2 h. Finally, the absorbance at 450 nm was measured using a Spectra Max M5 microplate reader (Molecular Devices, CA, USA).

### Cell cycle analysis

The cell cycle analysis was performed with cells seeded in 48-well plates (3 × 10^3^ cells/well) in growth media. The cells were allowed to attach overnight. The cultures were transduced with the AAV-CFs-FLAG vector and incubated for 72 and 96 h. The collected cells were then washed in PBS and fixed in 75% ethanol. The cells were stained with 50 μg/mL propidium iodide containing 10 μg/mL RNase A and then analyzed in a FACS Calibur flow cytometer (BD Biosciences, CA, USA). The percentages of cells in the G1, S, and G2-M phases were determined.

### Statistical analysis

The data are expressed as the mean ± SEM. The results were analyzed via one-way analysis of variance (ANOVA) (general linear model) using SPSS software (version 13.0). The positions of the samples on the plate were randomly assigned, and all samples were run in replicates of eight. Significant differences between treatments were tested using a Student’s t- test. Differences are reported at two significance levels, 0.05 or 0.01.

### Availability of supporting data

The mRNA-seq data were deposited at NCBI with the Gen Bank accession number KF833357 (http://www.ncbi.nlm.nih.gov/nuccore/KF833357).

## Abbreviations

OPM: Ovine primary myoblast
TGF-β: Transforming growth factor β
DMEM: Dulbecco’s modified Eagle’s medium
FBS: Fetal bovine serum
CDK: Cyclin-dependent kinase
PBS: Phosphate buffered solution
p21: Cyclin-dependent kinase inhibitor 1
Akt I: Alpha serine/threonine-protein kinase
Rb: Retinoblastoma
